# Application of Multi-Criteria Optimization Methods in the Calibration Process of Digital Measuring Instruments

**DOI:** 10.3390/s23062984

**Published:** 2023-03-09

**Authors:** Maciej Klebba, Arkadiusz Adamczyk, Mariusz Wąż, Dominik Iwen

**Affiliations:** 1 Faculty of Mechanical and Electrical Engineering Polish Naval Academy, Smidowicza 69, 81-127 Gdynia, Poland; 2 Faculty of Navigation and Naval Weapons Polish Naval Academy, Smidowicza 69, 81-127 Gdynia, Poland

**Keywords:** optimization, uncertainty, applied metrology, measurement methods

## Abstract

The article describes the use of multi-criteria optimization methods during the calibration of digital multimeters. Currently, calibration is based on a single measurement of a specific value. The aim of this research was to confirm the possibility of using a series of measurements in order to reduce the measurement uncertainty without significantly extending the calibration time. The automatic measurement loading laboratory stand used during the conducted experiments was essential to obtain results that allowed confirming the thesis. This article presents the applied optimization methods and the results of the calibration of sample digital multimeters obtained thanks to them. As a result of the research, it was found that the use of a series of measurements increased the accuracy of the calibration, reduced the measurement uncertainty, and shortened the calibration time compared to traditional methods.

## 1. Introduction

The calibration of measuring systems, as well as individual measuring instruments is required in many fields of technology. The requirement to perform regular calibration is described in many normative documents such as [1,2,3]. It is performed in laboratories in a precisely defined manner. Both the measurement procedures according to which the calibration is performed and the organization of the laboratory itself are strictly defined [4,5,6,7,8]. This allows an increase in the repeatability and reliability of the tests performed [9,10]. With the development of technology, more and more complex and accurate measuring devices are used. Many of the new devices require completely new calibration procedures [11,12]. On the other hand, work is still underway to improve the existing calibration methods [13,14]. More and more methods are based on semi-automated or fully automatic stations [15,16]. This allows both shortening the calibration time and eliminating at least some personal errors. During calibration, the main task is to determine the error with which the tested instrument made the measurement. This error can be estimated from a single measurement [17]. It can also be determined by taking a series of measurements and calculating the average value, which is more accurate, but also more time-consuming. In addition, the appropriate number of measurements in the series should be determined. A properly selected number of measurements can be described as “optimal”. What does optimal mean?

In many areas of human activity, the best use of resources is sought to obtain the maximum effect. The field of science supporting the making of this type of decision is optimization. Due to the number of criteria, optimization can be divided into:

- Single-criterion—defining one function that describes a specific problem and finds its extreme.
- Multi-criteria—finding the optimal solution that is appropriate from the point of view of each criterion.

The criterion is used to compare individual solutions [18].

Multi-criteria optimization methods are often used in economic, social, and technical sciences. The scope of their application is very wide. In the literature, there are examples of the use of optimization methods during titanium turning [19], transport issues [20], furniture production [21], traffic [22], humanitarian aid [23], energy distribution [24], logistic [25,26,27,28], etc. [29,30,31,32].

Currently, the procedures for calibrating digital multimeters are based on a single measurement for a specific value. This is an approach that has been used for years; however, there are situations in which the use of a single measurement does not allow for an accurate estimation of the measurement error. Often, according to the authors’ research, in more than 40% of cases, the results obtained using a digital multimeter are unstable. Although instability occurs most often at the level of the last digit, such a situation makes it necessary for the person performing the calibration to choose one of several possible measurement results. In order to avoid a situation where the efficiency of the device is decided on the basis of a randomly selected result, the authors propose changing the current approach.

It is possible to approach the problem differently.

In order to create a new method of calibrating multimeters, a digital multimeter calibration procedure was developed in which a single measurement was replaced with a series of measurements for each measured value. A proprietary laboratory stand for the automatic setting of values from the calibrator and automatic reading of measurement results was developed [17]. The question is how many measurements should be taken during the series to sufficiently increase the accuracy, reduce the uncertainty to an acceptable level, and at the same time, not significantly increase the calibration time. According to the authors, multi-criteria optimization methods are a perfect tool for determining the optimal number of measurements for a series. The following part of the article presents how to use the recently developed method and its benefits.

Section 2 of this article describes the calibration process and the multi-criteria optimization methods used in the research. Section 3 presents the method of performing the calculations for an example device using three optimization methods. Section 4 presents the results of the tests carried out on several different types of multimeters. Section 5 shows how the applied method affects the uncertainty of measurement and the change of the decision about the efficiency of the instrument.

## 2. Materials and Methods

### 2.1. Calibration

The calibration of measuring instruments consists of determining the errors obtained during the measurements of certain values of various physical quantities. These errors are calculated on the basis of a comparison of the value measured with the tested device and the reference value obtained from the control device. Measurements are made for strictly defined values of the tested physical quantities. The number and values of individual measurement points are specified in the calibration methodology. The methodologies are specified either by the manufacturer of the instrument or by the testing laboratory. During the full calibration procedure, depending on the type of instrument, it is necessary to make several to over 100 measurements of different values. The most-common method is measuring the same physical quantity with two instruments and comparing the obtained results. The first device is the one whose efficiency is tested. The second is a control device, the measurement uncertainty of which must be at least four-times lower than the tested instrument [33].

In the case of measurements with analog instruments, the presence of a human is practically always required to read the measurement result. The obtained results are compared with the readings from the reference instrument; the measurement error is determined; the measurement uncertainty is estimated; the correct operation of the measuring instrument is assessed. If a large number of measurements are required, this task is very time-consuming.

The procedure is similar for digital instruments. Digital instruments are often equipped with a communication interface that allows data to be transferred directly to a computer. Thanks to this solution, it is possible to partially or completely automate the measurements.

During calibration at a specific measurement point, it is possible to determine the error on the basis of a single measurement or on the basis of a series of measurements. The series of measurements consists of making a specified number of measurements of the same value of a physical quantity. The mean value after excluding gross errors is taken as the result of the series. The measurement series improves the quality of the obtained results and allows for a more reliable determination of the correct operation of the measuring instrument. With the increase in the number of measurements performed in the series, the result is closer to the real value, and the Type A uncertainty of such a measurement decreases. On the other hand, increasing the number of measurements increases the time and energy required to complete the entire calibration procedure.

**Figure 1:**
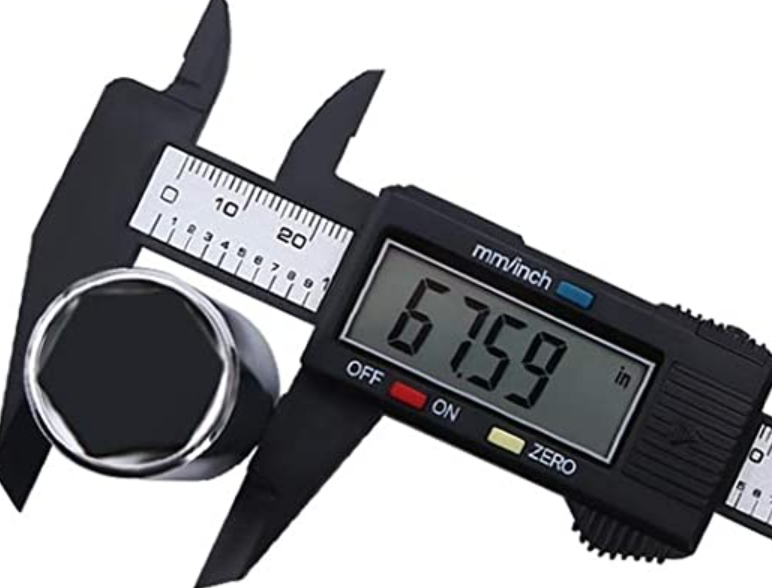
Measurement with a digital caliper [34].

This is where the problem begins: whether to evaluate an instrument on the basis of a single measurement, perform the number of measurements proposed in the calibration procedure, or estimate the optimal number of points in a series using multi-criteria optimization methods. At this point, one should look at the results obtained during the measurement with a digital instrument. The measurement with a digital instrument is performed at a certain resolution. The resolution is understood to be the smallest value indicated by the instrument. For example, the measurement of the outer diameter with a digital caliper is made at a resolution of 0.01 inches (Figure 1).

**Figure 2:**
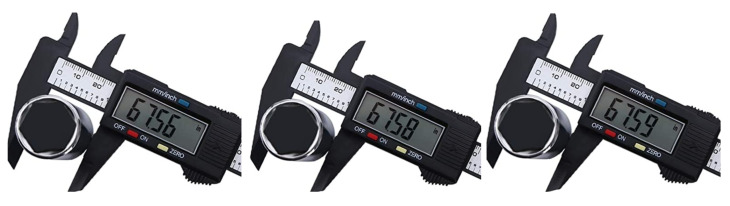
Three measurement results of the same value [34].

If the measurement is unambiguous and the result on the display does not change, the prediction of the efficiency on the basis of a single measurement is justified. However, it often turns out that, during the measurements, the indication of the digit in the resolution position is not constant. Changes from measuring the same value may change by one or more digits. In this case, determining the efficiency of the instrument on the basis of an ambiguous result is burdened with a large error (Figure 2).

During the research conducted on a group of over 150 digital instruments of various types, it turned out that measurement ambiguity occurred in over 40% of cases. Approximately 30% were changed by one value in the position of the last significant digit, and in 10% of the cases, the changes amounted to 2, 3, or more values in the position of the last significant digit.

On the basis of the obtained results, it was found that it is reasonable to carry out calibration on the basis of a series of measurements in order to avoid an error resulting from the ambiguity of the reading.

Some digital measuring devices, especially those with higher accuracy, are designed to be calibrated based on a series of measurements. In this case, the number of measurements in the series is determined in a constant manner in the methodology for all measurement points. As a standard, a series of measurements is used, consisting of 10 measurements, regardless of the measured value. The conducted research, the results of which will be presented later in the article, showed that the optimal number of measurements in a series is different for individual physical quantities. In this case, the efficiency of the calibration process can also be improved by changing the number of measurements in a series depending on the type of instrument and the measured physical quantity.

The main question is: What number of measurements in the series should be made to obtain a result that is sufficiently precise and, at the same time, does not cause a significant extension of the entire procedure in time? At this point, multi-criteria optimization methods should be used.

In the further part of the article, the use of three optimization methods will be presented. These methods allow both shortening the calibration time and increasing the accuracy of the obtained results.

### 2.2. Optimization

In technical issues and economics, sociology, or politics, the decision-maker faces the necessity to meet many needs, which in turn requires optimization in relation to many criteria. From this type of problem was born a discipline of mathematics called multi-criteria optimization. This discipline is used in a variety of subjects and under different names. In mathematics, it is called vector optimization and, in the political economy, “multi-criteria decision making”. In the literature, the terms Pareto optimization and poly-optimization are also often used. The basic concept in multi-criteria optimization is the Pareto solution, also known as the non-dominant solution, the effective solution, or the preferential solution.

Each technical, technological, or manufacturing process of a given product should constitute a compromise between the desire to ensure the product quality, increase its reliability, and reduce the cost of the materials, manufacturing, and operation costs or the time required to complete a given technology. Finding the most-favorable variant is possible after conducting a properly prepared optimization analysis. The optimization result largely depends on the formulation of the problem. It is particularly important to select criteria against which the entire process will be judged. In the optimization of technical processes, the most-frequently adopted criteria are:

- Performance criteria (functional, aesthetic);
- Technical criteria (general technical, manufacturing, material);
- Economic criteria (production costs and time, operating costs) [35].

It is difficult to find functional criteria in the calibration of measuring instruments, while technical and economic ones are perfectly justified. In order to carry out the full multi-criteria optimization process, three basic optimization criteria were adopted:

- The criterion related to the measurement error [f1(x)];
- The criterion related to the measurement uncertainty [f2(x)];
- The criterion of the total time of execution of the calibration [f3(x)].

All criteria described in detail in the further part of the article were developed on the basis of verified and recognized literature data. In addition, the authors’many years of experience in laboratory practice during calibration allowed for the proper selection and verification of the criteria. All considerations later in this article were based on the calibration of a digital multimeter (DMM).

#### 2.2.1. Criterion Related to the Measurement Error [$f_{1}\left(x\right)$]

The measurement error as defined in the document [36] is the measured quantity value minus a reference quantity value.

(1) $$\begin{array}{c} E=y_{i}-y_{k}+\delta y_{i}-\delta y_{k}, \end{array}$$

where:

*E*—measurement error;
$y_{i}$—an indication of the measuring instrument (*i* symbolizes successive measuring points);
$y_{k}$—value generated by the calibrator;
$\delta y_{i}$—instrument reading correction due to finite resolution;
$\delta y_{k}$—correction of the value generated by the calibrator, including the following factors:

- Drift since the last calibrator calibration;
- Changes caused by the effect of offset, non-linearity, and changes in the gain factor;
- Changes in ambient temperature;
- Supply voltage changes;
- Load effect resulting from the finite input resistance of the calibrated multimeter.

This is the classic approach when calibrating the multimeter with the assumption that, due to the specific resolution level of the digital multimeter indications, the scatter of the indicated values is not observed. As shown by the data presented in the previous section, this assumption is not met in over 40% of cases. Therefore, a modified Equation (1) was adopted for further research, replacing the value of a single measurement $y_{i}$ with a measurement series.

(2) $$\begin{array}{c} E=\bar{y_{i}}\left(x\right)-y_{k}+\delta y_{i}-\delta y_{k}, \end{array}$$

$\bar{y_{i}}\left(x\right)$—mean value from a series of *x* measurements.

For the optimization calculation, the objective function for the error criterion will contain only the absolute error computation. The components of the measurement equation describing the uncertainty will be included in the objective function related to the measurement uncertainty. During the calibration, the calculated error of measurement is the result. To optimize this process to improve its quality, one should strive to increase the precision of this error determination. Assuming that the average value of 30 measurements is a sufficient basis for estimating the actual error, the related optimization criterion should define how much we deviate from the actual value of the measurement error during the performance of individual measurement series. The objective function resulting from the absolute error takes the form:

(3) $$\begin{array}{c} f_{1}\left(x\right)=W-\left(\frac{\sum_{i=1}^{x}y_{i}}{x}\right), \end{array}$$

where:

*x*—the number of measurements in series;
$y_{i}$—the result of the i-th measurement;
*W*—the mean value of the result from 30 measurements.

#### 2.2.2. Criterion Related to the Measurement Uncertainty $\left[f_{2}\left(x\right)\right]$

When presenting the result of measuring a physical quantity and the real value, it is necessary to provide some quantitative information about the quality of this result, based on which its credibility can be estimated. Without such information, the measurement results can neither be compared with each other, nor with the reference values given in specifications or standards. It is commonly known that, when all known or expected error components are calculated and when they are made as appropriate corrections, there is still uncertainty as to the correctness of the result obtained and doubts as to how well the measurement result represents the value of the quantity resulting from the measurement [37]. To sum up, the given measurement result is complete only when it contains both the measurand value and the measurement uncertainty related to this value. The uncertainty of the measurement result consists of a series of components that can be grouped into two categories, according to how their numerical values are calculated:

A—those that have been calculated by statistical methods;
B—those that have been calculated by other methods.

The A method is used when it is possible to carry out many independent observations of one of the input quantities under the same measurement conditions. If the resolution of the measurement process is sufficient, the obtained results are characterized by a noticeable dispersion.

The B method is the determination of the uncertainty associated with the estimate $y_{i}$ and the input quantity $Y_{i}$ in a manner other than by statistical analysis of a series of measurements. The standard uncertainty $u(y_{i})$ is determined by an analysis based on all available information on the possible variability of $Y_{i}$. This information may include:

- Data from previous measurements;
- Experience and general knowledge of the behavior and properties of appropriate measuring instruments;
- Manufacturer specifications;
- Data obtained from calibration certificates or other certificates;
- Uncertainties related to reference data obtained from the literature.

The components of the standard uncertainty involved in the uncertainty budget are:

$u(y_{k})$—the uncertainty related to the calibration of the standard, estimated on the basis of the records of the last calibration certificate;
$u(\delta y_{i})$—uncertainty related to the resolution of the calibrated multimeter;
$u(\delta y_{k})$—the uncertainty related to the factors affecting the quantity generated by the calibrator, estimated from the manufacturer’s data;
$u(\bar{y_{i}}\left(x\right))$—uncertainty related to the dispersion of the series of measurements.

The general form of the formula for standard uncertainty is [36]:

(4) $$\begin{array}{c} u(y)=\sqrt{\sum_{j=1}^{N}c_{j}^{2}u_{j}^{2}\left(x\right)}, \end{array}$$

where:

*j*—successive component of uncertainty;
*N*—number of uncertainty components;
*c*—sensitivity coefficient.

For the calibration of a digital multimeter, assuming that the desired quantities are not correlated with each other and the sensitivity coefficients take the value 1 or −1 will take the form [36]:

(5) $$\begin{array}{c} u\left(y\right)=\sqrt{\left(u{\left(y_{k}\right)}^{2}+u{\left(\delta y_{i}\right)}^{2}+u{\left(\delta y_{k}\right)}^{2}+u{\left(\bar{y_{i}}\left(x\right)\right)}^{2}\right)} \end{array}$$

As recommended in [36,37], the uncertainty in the laboratory is reported not as the standard uncertainty, but as the expanded uncertainty *U*. This uncertainty is obtained by multiplying the estimated standard uncertainty u(y) by the coverage factor *k* [36]:

(6) $$\begin{array}{c} U=k\cdot u(y) \end{array}$$

When performing the calibration, the expansion factor is taken as the value k=2, which corresponds to a normal distribution, or k=1.65, which corresponds to a rectangular uncertainty distribution. When developing the uncertainty budget, it turns out that, sometimes, some of the dominant components may have a rectangular distribution. In other cases, the dominant distribution is the normal distribution. The determination of the parameter *r* in the proposed method is based on the fact that the distribution of the output quantity converges to the distribution of the PN (PN distribution—a distribution that is the convolution of a single normal distribution and a single rectangular distribution). The parameter *r* of the PN distribution is determined by the measured component of the rectangular distribution in all components of the uncertainty budget. The method approximates the unknown coefficient of expansion by a factor for distribution: normal, trapezoidal, and rectangular [38].

(7) $$\begin{array}{c} U\left(y\right)=\sqrt{\frac{3}{r^{2}+1}\left(1+r-2\sqrt{r(1-p)}\right)}\cdot \\ \sqrt{\sum_{i=1}^{N}{\left(\frac{\partial f}{\partial x_{i}}\right)}^{2}u_{i}^{2}\left(x_{i}\right)}, \end{array}$$

where:

*p*—confidence level.

The parameter *r* is determined by the formula [38]:

(8) $$\begin{array}{c} r=\frac{u_{i}\left(y\right)}{\sqrt{u_{c}^{2}\left(y\right)-u_{i}^{2}\left(y\right)}}, \end{array}$$

where $u_{i}\left(y\right)$ is the largest share in the uncertainty of the composite input quantity with a rectangular distribution and $u_{c}\left(y\right)$ is the combined standard uncertainty. Detailed Equation (7) will be used for calculations, and the utility function determined on its basis takes the form:

(9) $$\begin{array}{c} f_{2}\left(x\right)=\sqrt{\frac{3}{r^{2}+1}\left(1+r-2\sqrt{r(1-p)}\right)}\cdot \\ \sqrt{u{\left(y_{k}\right)}^{2}+u{\left(\delta y_{i}\right)}^{2}+u{\left(\delta y_{k}\right)}^{2}+u{\left(\bar{y_{i}}\left(x\right)\right)}^{2}} \end{array}$$

#### 2.2.3. Criterion of the Total Time of Execution of the Calibration $\left[f_{3}\left(x\right)\right]$

When performing the calibration for one measuring point, the time to complete the full procedure is shorter than in a measuring series. Thanks to the solution that allows for automatic reading of the measurement result, this time is significantly shortened, and it is possible to perform a measurement series in a time close to or shorter than a single measurement in manual mode. For optimization calculations, an objective function should be formulated that includes the relationship between the number of measurements performed in a series and the total time of the complete calibration procedure. In the case of the calibration of digital multimeters, this function takes the form:

(10) $$\begin{array}{c} f_{3}\left(x\right)=t_{0}+\left(\left(t_{s}\cdot x\right)-t_{s}\right)p_{p}, \end{array}$$

where:

$t_{0}$—automatic calibration time with one measurement;
$t_{s}$—time of the next measurement in the series;
$p_{p}$—total number of measurement points.

The adequate area of research is usually determined due to the technical possibilities of research stands and the nature of the phenomena and processes under study. In the case of the optimization of the calibration process, the variable parameter in all defined objective functions is the number of measurements performed in the series. The limitations of the possible values that this parameter can take result from the nature of the calibration itself. The result is the measurement error and the uncertainty of its determination. Considering that making at least three readings prevents coarse errors, this is the minimum number of measurements in the series. Correct results are assumed if the readings are uniform within the random error limits. The probability of a coarse error in the two readings is $p_{1}^{2}$ and in three readings is $p_{1}^{3}$, where $p_{1}$ is the probability of a coarse error with one reading. Hence, according to [39], the recipe for the correct reading of the results is as follows: after bringing the reference quantity to the instrument to be calibrated, wait time *t*, then take n readings at intervals $t_{s}$ and take the average value of *n* readings as the result. Due to the assumption that the methodology described in the paper is to shorten the time of performing the calibration, the maximum number of measurements performed in a series should be assumed. In order to generalize the considerations, the permissible variability of the number of measurements in the series within the range was assumed 3<x<30.

#### 2.2.4. Multi-Criteria Optimization Methods

In single-criteria optimization problems, the objective function returns one specific min or max value. In practice, it is difficult to formulate a single-criterion task as it is often necessary to take into account a whole set of various conditions. Among the many methods of multi-criteria optimization, the following seem to be the most useful for solving technical problems:

- Weight objectives method;
- Hierarchical optimization method;
- Trade-off method;
- Global criterion method;
- Method of distance functions;
- Min–max method;
- Goal programming method.

In order to check whether the assumptions presented in the above part of the article are correct, three methods were selected for practical verification (weight objectives, min–max, and method of distance function). Other methods will be tested in further stages of research [40].

## 3. Result

In order to check whether the use of a series of measurements with the help of an automated calibration stand will allow improving the quality of the obtained results, a series of tests were carried out using various types of digital multimeters. The research was carried out in the years 2018–2020, and multi-criteria optimization methods were used to determine the optimal number of measurements in the series.

The first step was to perform optimization calculations in accordance with the previously presented principles. These calculations were designed to determine the number of measurements in a series appropriate for the selected criteria for each type of multimeter. Considering that the proposed solution is planned for practical implementation in everyday laboratory practice, the results obtained for individual measurement points were generalized for the entire calibration procedure of a given type of instrument. This article presents the test results based on the example of the FLUKE 27 digital multimeter with Serial Number 96000029.

To determine the optimal number of measurements performed in series, the criteria described above should be taken into account. The random nature of the obtained results, which are also the input data for the optimization calculations, necessitates some modification of the optimization methods. The classic approach consisting of carrying out calculations once and determining optimal Pareto solutions is insufficient in calibration. The calculations carried out in this way make it possible to determine the optimal number of measurements for one specific set of input data. The tests showed that, during the device’s test, the obtained results of measuring the same value of a given physical quantity, although similar, differed from each other. Thus, for each data set, the results of the optimization calculations may differ from each other. An example of the results obtained during a series of 10 measurements performed with the FLUKE 27 multimeter connected to the FLUKE 5500 calibrator for 40 V DC is presented in Table 1.

The data in Table 1 show that the results obtained with the same measurement tool showed a certain spread. This dispersion, despite the preserved character of the rectangular distribution, was different each time. For the calculations, a Visual Basic program was written, which works with a spreadsheet, in which the results of a series of 30 measurements were randomly simulated. For the input data obtained in this way, optimization calculations were carried out according to the rules of a given method. The result was saved in the correct cell, and then, another set of input data was generated, then the calculations were repeated. The entire procedure was repeated a certain number of times. During the research, it was assumed that the procedure would be repeated 1000 times. According to the decision-maker, from the set of 1000 Pareto-optimal solutions obtained in this way, one should choose the most-appropriate one. Due to the fact that the proposed solution is to be applied in practice, the way of making decisions should be simplified as much as possible. Therefore, it was assumed that the most-appropriate solution would be the solution that would occur the most significant number of times. For each measurement point, a sheet was created to perform the calculations simultaneously for all selected optimization methods. The adopted procedure for performing the simulation calculations resulted from the assumption of a random distribution of the input data. For the results of the calculations made with different optimization methods to be compared with each other, it is obvious that they must be performed on the same set of input data. In order to test the effectiveness of the proposed solution, three methods were used.

### 3.1. Weight Objectives Method

The method consists of reducing multi-criteria optimization to single-criteria optimization by introducing a substitute criterion, which is a weighted sum of the criteria [40]:

(11) $$\begin{array}{c} F\left(x\right)=\sum_{i=1}^{k}w_{i}f_{i}\left(x\right), \end{array}$$

where:

*k*—number of objective criteria;
*x*—number of measurements in series;
$w_{i}$—weights such that:

(12) $$\begin{array}{c} w\in \left[0,1\right]\;and\sum_{i=1}^{k}w_{i}=1 \end{array}$$

As a result of such transformation, we obtained a single objective function F(x), which we optimized using methods typical for a single-criterion task. The choice of criteria weights is problematic in this method, which obviously may lead to different solutions. For the calibration of DMMs, the objective function will be

(13) $$\begin{array}{c} F\left(x\right)=w_{1}f_{1}\left(x\right)+w_{2}f_{2}\left(x\right)+w_{3}f_{3}\left(x\right), \end{array}$$

where the objective functions are defined by Formulas (3), (9), and (10). Equation (12) takes the form:

(14) $$F\left(x\right)=w_{1}\left(W-\left(\frac{\sum_{i=1}^{x}p_{i}}{x}\right)\right)+\;w_{2}\sqrt{\frac{3}{r_{u}^{2}+1}\left(1+r_{u}-2\sqrt{r_{u}\left(1-p\right)}\right)}\cdot \;\sqrt{{u(y_{k})}^{2}+{u(\delta y_{i})}^{2}+{u(\delta y_{k})}^{2}+{u(\bar{y_{i}}(x)}^{2}}+\;w_{3}\left(t_{0}+\left(t_{s}-t_{s}\right)p_{p}\right)$$

Due to the random nature of the obtained results for a single measurement point, the method based on the simulation described above was used. For a specific measurement point, a spread of one or two digits in the last position of the display was assumed. Thirty results within the assumed range of variability were generated randomly. For the adopted data set, calculations were performed in accordance with the rules applicable in the weighted objectives method. For this purpose, the minimum value of the objective function from Equation (14) was calculated for each value of *x*, where 3<x<30. For the purposes of the article, the weighting factors at the level were adopted:

(15) $$\begin{array}{c} w_{1}=0,1;\;w_{2}=0,4;\;w_{3}=0,5. \end{array}$$

The weight factor values were selected in such a way so as to place the greatest emphasis on the calibration time and measurement uncertainty. These values were adopted arbitrarily by the authors.

The weighting factors determination is valid if all the objective functions are expressed in the same units and with the same order of magnitude. In actual calculations, such a comfortable situation usually does not occur. In order to eliminate the influence of various units, in which particular functions’ criteria are typically expressed, it is aimed to ensure that all functions result in the calculation of an order of magnitude of a similar order. Only then does the objective function make sense. For this reason, the objective functions were assumed in the normalized form for the calculations:

(16) $$\begin{array}{c} F\left(x\right)=\sum_{i=1}^{k}w_{i}\frac{f_{i}\left(x\right)}{|f_{i}^{0}|}, \end{array}$$

where:

$f_{i}^{0}=-minf_{i}\left(x\right),\;when-minf_{i}\left(x\right)⩾maxf_{i}\left(x\right)$;
$f_{i}^{0}=maxf_{i}\left(x\right),\;when-minf_{i}\left(x\right)<maxf_{i}\left(x\right)$;

for every *x* in an adequate common area.

**Figure 3:**
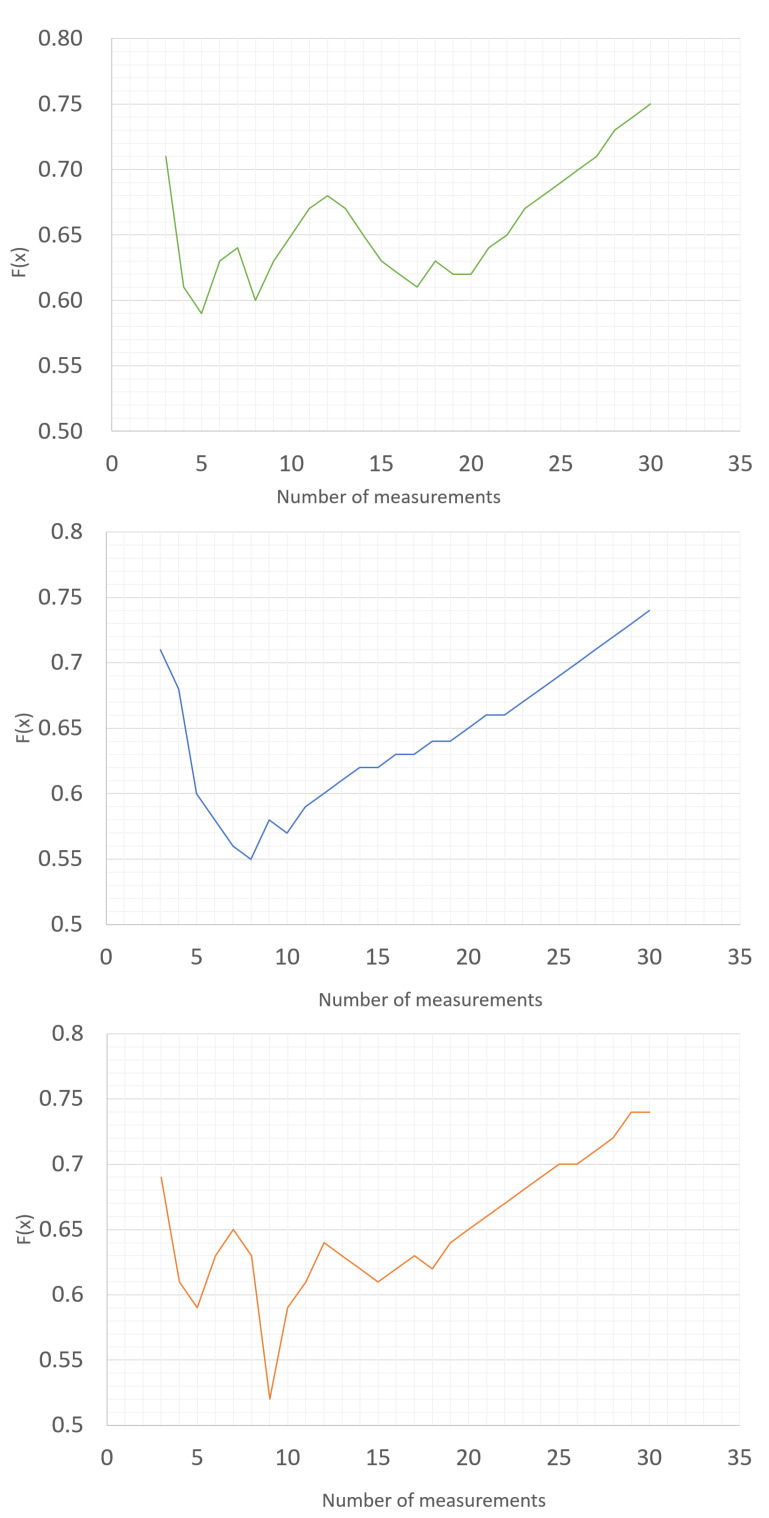
Optimal solutions in the weighted objectives method for different sets of input data.

In this way, the calculated minimum of the objective function determines the optimal number of measurements in the series for a given type of instrument and for a specific measurement point. It should be remembered that the obtained number is optimal for a specific set of 30 random input data. For a different dataset, this value may change. In order to perform the calculations and simulations, a program in Visual Basic was written based on the MS Excel spreadsheet. The results obtained for the three exemplary input data sets obtained during the simulation of the 40V DC test point for the FLUKE 27 multimeter are shown in Figure 3.

**Figure 4:**
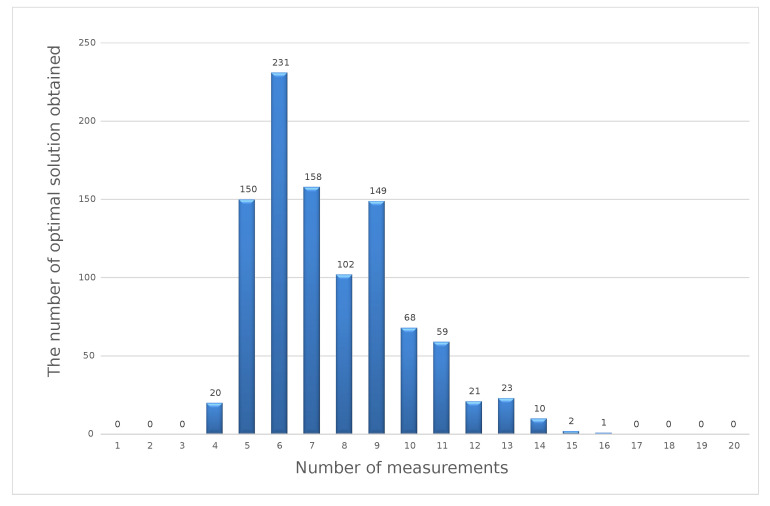
Optimal solutions in the weighted criteria method for a simulated series of 1000 sets of input data.

The graphs presented below show that the results of the optimization calculations for individual sets of input data may differ. For four input data sets, the optimal solution turned out to be the number of measurements in the series at a level of 5, 6, 7, or 9. For the specified number of measurements in the series to be a value that reflects the optimal number for different possible values of the measurement results, the calculation was performed 1000 times. Each time, we redrew the results from a specific range of variability. As a result of the simulation, we obtained 1000 results determining the optimal number of measurements in a series for a specific measuring point. The sample results obtained from the simulation are presented in the form of a histogram in Figure 4. The optimal solution is the result that was repeated the greatest number of times.

The histogram presented above shows that, for the 40V DC measuring point, the most-frequently obtained result of optimization calculations using the weighted criteria method was six measurements in a series. The presented calculation scheme should be repeated for each measuring point specified in the calibration methodology for a given type of measuring instrument.

### 3.2. Method of Distance Functions, Min–Max Method

**Figure 5:**
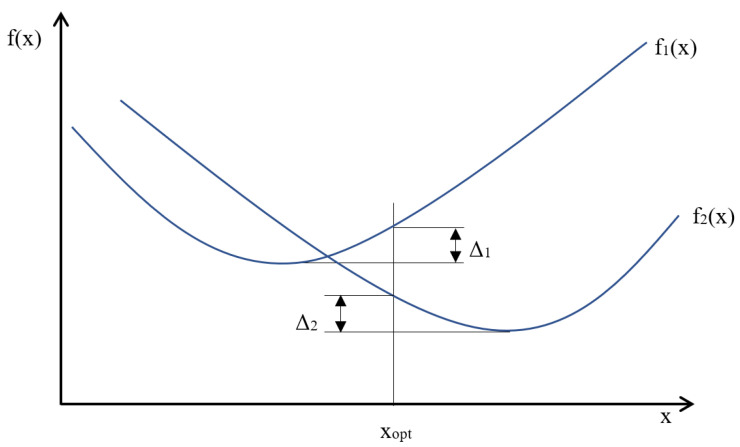
Pareto optimum example for two functions of one variable.

The necessary condition for determining the optimal solution is the definition of the Pareto optimum [41]. According to the definition, the solution is optimal when determining another one cannot improve the value of any criterion function without deteriorating any other criterion functions’ value. An example of a Pareto optimum for two functions of one and the same variable is shown in Figure 5.

The min–max method consists of minimizing the maximum deviations from the extreme values (optimal solutions) for all functions of the optimization criteria. The formulas give the relative deviation for each *i* criterion function:

(17) $$\begin{array}{c} \Delta_{i}^{\prime }\left(x\right)=\frac{|f_{i}\left(x\right)-F_{i}^{0}|}{|F_{i}^{0}|} \end{array}$$

(18) $$\begin{array}{c} \Delta_{i}^{\prime \prime }\left(x\right)=\frac{|f_{i}\left(x\right)-F_{i}^{0}|}{|f_{i}^{0}|} \end{array}$$

for $F_{i}^{0}\neq 0$ and $f_{i}^{0}\left(x\right)\neq 0$ released from the dimensionality of the objective functions, which for the minimized objective functions determine the relative increments of the function’s value, and for the maximized objective functions, they determine the relative decreases in the value of these functions.

In the min–max method, one searches for such parameter values for which individual objective functions give the values of the result parameters that are the same while, at the same time, as small as possible, from the extremes of both objective functions (approximate solution), i.e., those for which the increases and the decreases of both objective functions are the same and as small as possible [42].

Use the formula for the calculations:

(19) $$\begin{array}{c} \sum_{i=1}^{k}{\left[{\left(\frac{|f_{i}\left(x\right)-F_{i}^{0}|}{|F_{i}^{0}|}\right)}^{P}+{\left(\frac{|f_{i}\left(x\right)-F_{i}^{0}|}{|f_{i}^{0}|}\right)}^{P}\right]}^{\frac{1}{P}}\to MIN \end{array}$$

If the exponent P=2 is used in the calculation, the distance between the approximate and optimal solution is minimized, i.e., the distance function method will be implemented. Increasing the value of the exponent to P=∞ leads to the min–max method, i.e., minimization of the maximum deviations of the optimal solution from the approximate one.

The value of the exponent from Equation (19) was assumed at the level of P=100.

**Figure 6:**
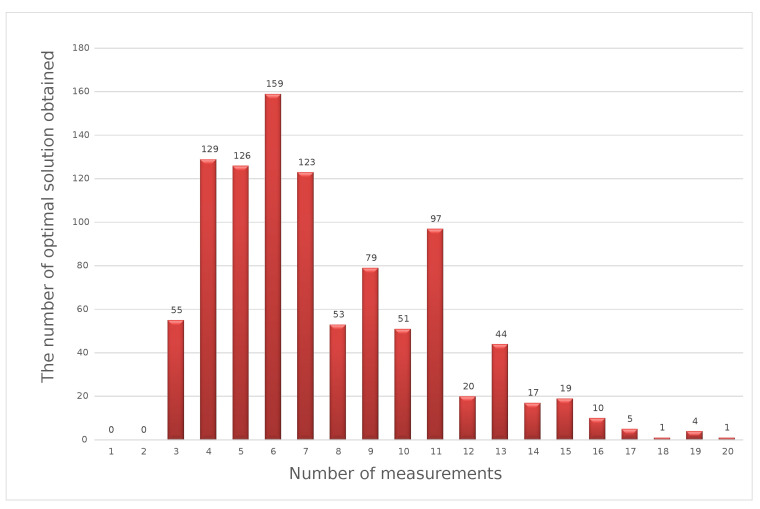
Optimal solutions in the min–max method for a simulated series of 1000 input data sets.

The results of the exemplary calculations for the min–max method and the same set of input data as for the weighted criteria method are presented in Figure 6.

**Figure 7:**
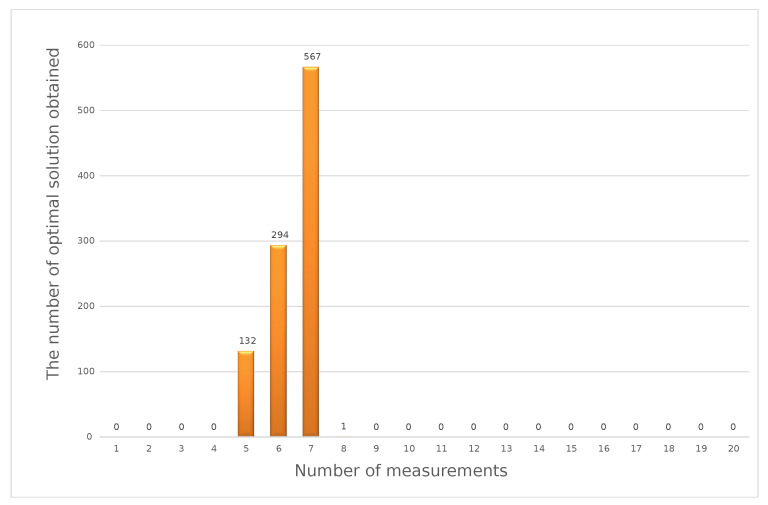
Optimal solutions with the distance function method for a simulated series of 1000 sets of input data.

The histogram presented above shows that, for the 40 V DC measuring point, the most-frequently obtained result of the optimization calculations using the weighted criteria method was six measurements in a series. To perform calculations using the distance function method, the value of the exponent *P* = 2 should be assumed. Similar to the previous method, calculations should be made according to Formula (19). Sample results obtained for the same set of input data as in the previous methods are shown in Figure 7.

The histogram presented above shows that, for the 40V DC measuring point, the most-frequently obtained result of optimization calculations using the weighted criteria method was seven measurements in a series. The nature of the distribution of the obtained results allows for an unambiguous statement of which variant occurs most often, and this one was determined as the optimal solution according to the method used. Practice has shown that the results obtained with different methods may differ from each other. In the considered example, the results for the three methods used are:

x=6—for the weight objectives method;
x=6—for the min–max method;
x=7—for the distance function method.

**Figure 8:**
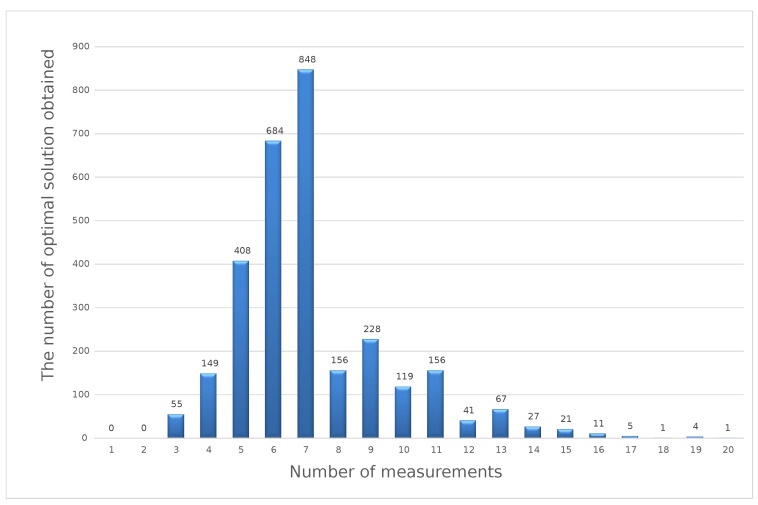
The result of optimization calculations with three methods for the point 40 V DC.

As during the calibration, one value of the number of measurements in the series should be determined, the obtained results for individual methods were added up, and the most-frequently repeated value obtained in this way was taken as the optimal (Figure 8).

This is, of course, the optimal solution for a specific measuring point. In the considered example, it is the value of the number of measurements in the series for the 40 V DC test point. The simulations should be performed for all measuring points in a given type of measuring instrument in the calibration procedure. In the considered example, in accordance with the procedure applicable at the time of writing the thesis at the 1st Military Metrology Center in Gdynia, the number of measuring points was 55.

## 4. Practice

In order to compare the proposed methodology to the currently used method, simulation calculations were made for all measuring points of several types of multimeters calibrated at the 1st Military Metrology Center. Detailed results obtained using an automated measuring station are presented in the example of the calibration of a FLUKE 27 multimeter. The basis for determining the measuring points was the procedure PP-07.10.01-2-2018-1WOM [43]. The simulation calculations were performed using the three methods described above:

- Weight objectives method;
- Min–max method;
- Distance function method.

The optimal number of measurements in a series depends on the parameter being measured and the level of variability of the last digit of the measured result. The research carried out on various types of multimeters showed that the most-common situation will be variation by one value at the level of the last digit. Therefore, to determine the optimal number of measurements in the series, the simulation results obtained for assuming a variability by one value were adopted.

For the specified number of measurements in a series, a calibration protocol was created, and the automated calibration of the instrument was carried out. Then, manual calibration was performed by making a single measurement at the same points. Calibration was performed with the same standard calibrator by three different people. Another method was to use the standard procedure in the MET/CAL program [44]. This procedure, like manual calibration, is based on a single measurement at each point. This calibration also was performed with the same standard calibrator by three different people. The effects obtained as a result of process optimization and automation can be described as two basic ones:

- Shortening the calibration time;
- Improving the quality of the results obtained.

**Figure 9:**
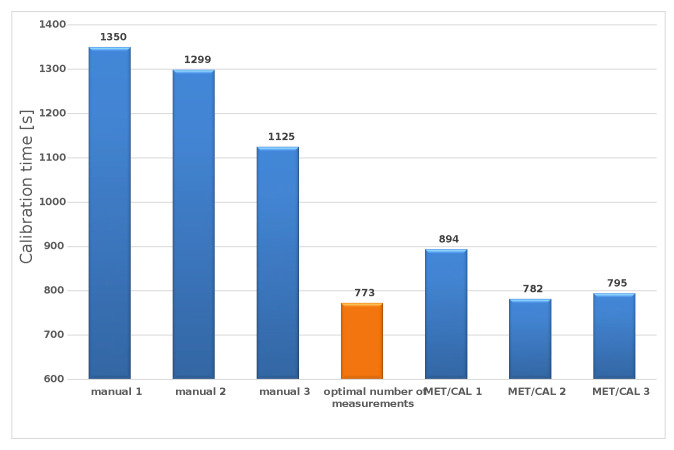
The result of optimization calculations with three methods for the point 40 V DC.

The shortening of the calibration time of the FUKE 27 multimeter was on average 38% compared to the manual calibration performed for one measurement at each point. The time reduction compared to the currently used semi-automatic method based on the MET/CAL software and a single measurement at each point was on average 6% (Figure 9).

Please note that manual calibration, as well as calibration using the MET/CAL software were performed based on one measurement (*x* = 1). Automatic calibration using the optimal number of measurements was performed by making seven measurements in a series (*x* = 7).

**Figure 10:**
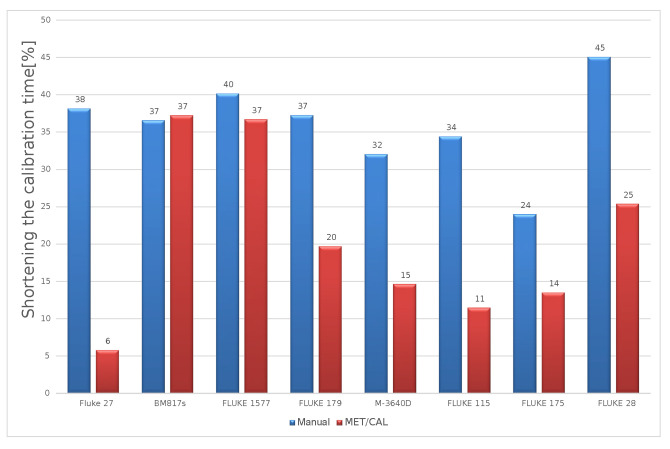
Reduced calibration time.

Similar studies were also carried out for other types of instruments. The obtained results confirmed the effectiveness of the method. The graphs show the results for seven sample multimeters. The reduction of the calibration time compared to manual calibration was 24% to 45%. On the other hand, compared to the semi-automatic method based on the MET/CAL software, the time reduction ranged from 6% to 37% (Figure 10).

**Figure 11:**
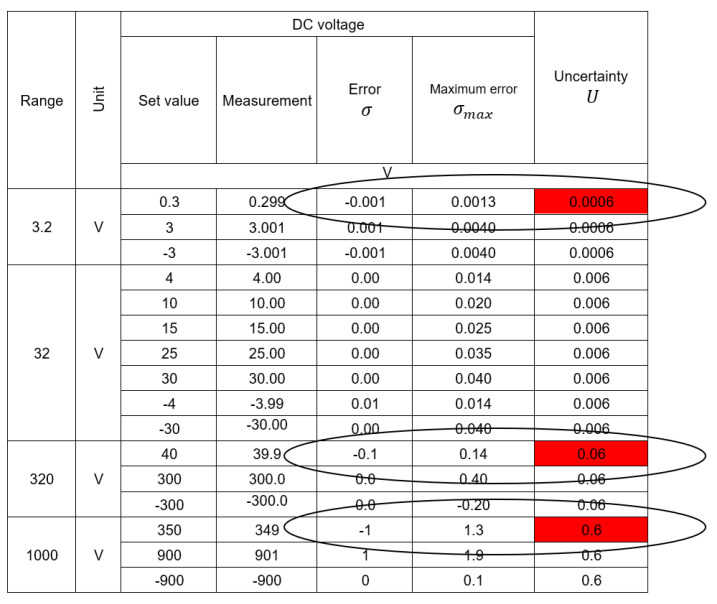
Manual calibration result for the point 40 V DC.

The second element of utilization as a result of the optimization is maintaining or improving the quality of the obtained measurement results. Quality is understood as the accuracy of determining the real value of the measured quantity and the uncertainty of the obtained result. In the case of a single measurement result, the obtained value is correct under the condition of a stable and unchanging reading from the instrument. As the results from the data presented in the first part of the article, this situation does not occur in over 40% of the measurements. A better approximation of the actual measured value is the average value from the series of measurements. In order to illustrate the influence of the measurement series on the calibration result, an example from the protocols presented in Figure 11 can be used.

**Figure 12:**
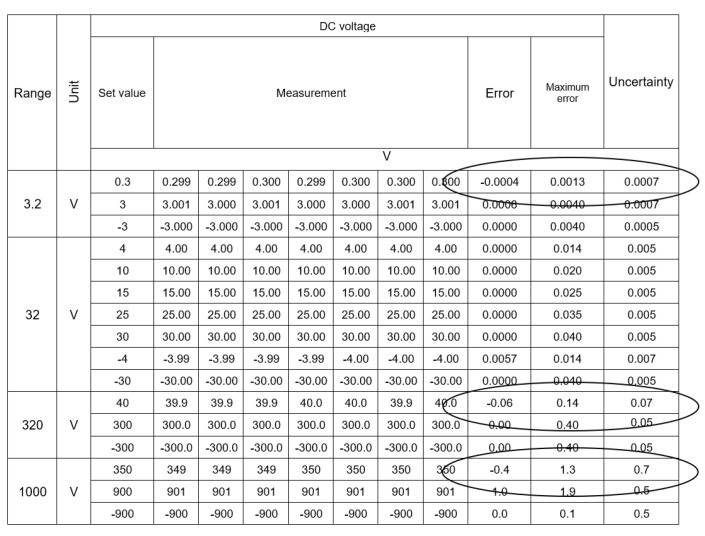
The result of optimization calculations with three methods for the point 40 V DC.

In the measurement points marked in the tables, thanks to the use of a series, the obtained result, being the average of seven measurements, differed from the values obtained during a single measurement. Thus, despite a slight increase in measurement uncertainty, which was caused by the addition of an additional factor to the uncertainty budget resulting from the standard deviation, the sum of the error and expanded uncertainty did not exceed the permissible error in any of the previously marked points. According to the valid rules of adjudication of conformity [7,8], this is Case 2 when the calibrator cannot decide about the conformity of the instrument with the requirements. It causes the necessity to calculate new permissible error limits or change the class of the measuring device in the measuring ranges in which this case occurred. The use of the proposed method of automatic calibration with a series of measurements allows for obtaining the results presented in Figure 12. In the measurement points marked in the tables, thanks to the use of a series, the obtained result, being the average of seven measurements, differed from the values obtained during a single measurement. Thus, despite a slight increase in measurement uncertainty, which was caused by the factor added to the uncertainty budget resulting from the standard deviation, the sum of the error and expanded uncertainty did not exceed the permissible error in any of the previously marked points.

## 5. Conclusions

The calibration of digital instruments is a field that is constantly evolving. The possibility of using partially or fully automatic stations allows for significant simplification, lowering measurement uncertainty, and speeding up the calibration process. The article presented a proposal to replace the currently used method of calibrating digital multimeters based on a single measurement with a series of measurements. The automatic measurement loading laboratory stand used during the conducted experiments enhanced the entire calibration procedure. Of course, both the time and the accuracy of the obtained results strongly depend on the assumed number of measurements in the series. Therefore, a mathematical tool, which is represented by the multi-criteria optimization methods, was used. The multi-criteria optimization method allows for determining the optimal number of measurements in a series for a specific type of instrument. Optimization calculations are only required when developing a new calibration procedure. The three methods presented in the article (weight objectives, min–max, and method of distance function) will be expanded in further work, in order to check their suitability in the proposed method. The tests carried out on several different types of multimeters, presented in this article, initially confirmed the effectiveness of the proposed method. Currently, the method is being tested on a wide group of instruments at Polish Military Metrology Centers. We believe that the proposed new approach to the calibration of digital multimeters will be implemented in subsequent laboratories.

## Figures and Tables

**Table 1:** Series of 10 measurements performed with the FLUKE 27 multimeter connected to the FLUKE 5500 calibrator for 40 V DC.

Measurement	Series 1	Series 2	Series 3	Series 4	Series 5
**1**	40.1	40.1	40.1	40.1	40.0
**2**	40.0	40.1	40.1	40.0	40.1
**3**	40.1	40.1	40.0	40.1	40.0
**4**	40.0	40.1	40.0	40.0	40.1
**5**	40.1	40.1	40.1	40.1	40.0
**6**	40.1	40.1	40.1	40.1	40.1
**7**	40.0	40.0	40.1	40.0	40.0
**8**	40.0	40.1	40.0	40.0	40.1
**9**	40.1	40.1	40.1	40.0	40.0
10	40.1	40.0	40.1	40.0	40.1
**Average**	40.06	40.08	40.07	40.04	40.05

## Data Availability

Not applicable.

## References

[1] (2018). 2018-02-General Requirements for the Competence of Testing and Calibration Laboratories.

[2] National Institute of Standards and Technology (2019). Selected Laboratory and Measurement Practices and Procedures to Support Basic Mass Calibrations.

[3] (2007). Guidelines for the Determination of Calibration Intervals of Measuring Instruments.

[4] Geronimo B.M., Lenzi G.G. (2023). Maturity Models for Testing and Calibration Laboratories: A Systematic Literature Review. Sustainability.

[5] Stajkovic A., Igic N.K.D.D., Krstic I. (2021). Improving the quality of environmental testing through the implementation of ISO 17025 standards. Facta Univ..

[6] (2021). Guideline for Describing Scopes of Accreditation.

[7] (2019). Guidelines on Decision Rules and Statements of Conformity.

[8] (2021). Guidelines for Measurement Uncertainty in Testing.

[9] Oliveira da Silva F.M., Silverio K.S., Castanheira M.I., Raposo M., Imaginário M.J., Simões I., Almeida M.A. (2022). Construction of Control Charts to Help in the Stability and Reliability of Results in an Accredited Water Quality Control Laboratory. Sustainability.

[10] Piwowar-Sulej K., Rojek-Nowosielska M., Sokołowska-Durkalec A., Markowska-Przybyła U. (2022). Maturity of CSR Implementation at the Organizational Level—From Literature Review to a Comprehensive Model. Sustainability.

[11] Yan P., Zhang W., Yang L., Zhang W., Yu H., Huang R., Zhu J., Liu X. (2023). Online Calibration Study of Non-Contact Current Sensors for Three-Phase Four-Wire Power Cables. Sensors.

[12] Tran C.-S., Hsieh T.-H., Jywe W.-Y. (2021). Laser R-Test for Angular Positioning Calibration and Compensation of the Five-Axis Machine Tools. Appl. Sci..

[13] Krajewski M., Sienkowski S. (2012). Computer software for calibration digital multimeters and calibrators. Electr. Rev..

[14] Makowski P., Piróg P. (2012). Automation of measuring installation for calibration of decade resistor at the Central Military Calibration Laboratory. Bull. Mil. Univ. Technol..

[15] Patonis P. (2023). Methodology and Tool Development for Mobile Device Cameras Calibration and Evaluation of the Results. Sensors.

[16] Leizea I.H., Puerto P. (2023). Calibration Procedure of a Multi-Camera System: Process Uncertainty Budget. Sensors.

[17] Grzeczka G., Klebba M. (2020). Automated Calibration System for Digital Multimeters Not Equipped with a Communication Interface. Sensors.

[18] Oswald M. (2005). Basics of Structure Optimization.

[19] Zawora J., Marciniak M., Dąbrowski L. (2016). Multi-criterion optimization of the titanium turning. Mechanik.

[20] Malesa A. (2012). Multi-criteria optimization as applied to transport issues. WSEI Sci. Pap. Transp. Inform. Ser..

[21] Kłosowski G., Kozłowski E. (2017). Use of multicriterial optimization in furniture manufacturing process. IapgoŚ.

[22] Gutjahr W., Nolz P. (2016). Multicriteria optimization in humanitarian aid. Eur. J. Oper. Res..

[23] Stefanovic A., Stefanovic J., So J., Ostojic M. (2015). Multi-criteria optimization of traffic signals: Mobility, safety, and environment. Transp. Res. Part C Emerg. Technol..

[24] Buoro D., Casisi M., Nardi A.D., Pinamonti P., Reini M. (2013). Multicriteria optimization of a distributed energy supply system for an industrial area. Energy.

[25] Aljohani K. (2023). Optimizing the Distribution Network of a Bakery Facility: A Reduced Travelled Distance and Food-Waste Minimization Perspective. Sustainability.

[26] Gaggero M., Tonelli F. (2021). A two-step optimization model for the distribution of perishable products. Networks.

[27] Lin D., Zhang Z., Wang J., Yang L., Shi Y., Soar J. (2019). Optimizing urban distribution routes for perishable foods considering carbon emission reduction. Sustainability.

[28] Amin-Tahmasbi H., Sadafi S., Ekren B.Y., Kumar V. (2022). A multi-objective integrated optimisation model for facility location and order allocation problem in a two-level supply chain network. Ann. Oper. Res..

[29] Ma Z., Wang Y. (2019). Evolutionary constrained multiobjective optimization: Test suite construction and performance comparisons. IEEE Trans. Evol. Comput..

[30] Zhu Q.Z., Lin Q. (2020). HA constrained multiobjective evolutionary algorithm with detect-and-escape strategy. IEEE Trans. Evol. Comput..

[31] Ma Z., Wang Y. (2021). Shift-based penalty for evolutionary constrained multiobjective optimization and its application. IEEE Trans. Cybern..

[32] Liu Z.Z., Wang Y. (2019). Handling constrained multiobjective optimization problems with constraints in both the decision and objective spaces. IEEE Trans. Evol. Comput..

[33] International Organization of Legal Metrology (2013). International Vocabulary of Terms in Legal Metrology (VIML).

[34] https://narzedziownia.shop/.

[35] Płonka S. (2017). Multi-Criteria Optimization of Machine Parts Manufacturing Processes.

[36] European Accreditation Laboratory Committee (2013). Evaluation of the Uncertainty of Measurement in Calibration.

[37] Joint Committee for Guides in Metrology (2008). Evaluation of Measurement Data—Guide to the Expression of Uncertainty in Measurement.

[38] Fotowicz P. (2001). Distribution approximation principle for measurement result in calibration. Meas. Autom. Robot..

[39] Kostyrko K., Piotrowski J. (2021). Calibration of Measuring Equipment.

[40] Odu G.O., Charles-Owaba O.E. (2013). Review of Multi-criteria Optimization Methods—Theory and Applications. IOSR J. Eng..

[41] Linkov I., Varghese A., Jamil S., Seager T.P., Kiker G., Bridges T. (2004). Multi-criteria decision analysis: A framework for structuring remedial decisions at the contaminated sites. Comparative Risk Assessment and Environmental Decision Making.

[42] Milic J.K., Lukas M. (2023). Min–Max Optimal Control of Robot Manipulators Affected by Sensor Faults. Sensors.

[43] Euramet (2011). Guidelines on the Calibration of Digital Multimeters.

[44] Kubiszyn P. (2021). Guardband methods used to evaluate the results of digital multimeters calibration on the example of FLUKE MET/CAL software. Electrotech. Rev..

